# Chitin and Chitosan Preparation from Marine Sources. Structure, Properties and Applications

**DOI:** 10.3390/md13031133

**Published:** 2015-03-02

**Authors:** Islem Younes, Marguerite Rinaudo

**Affiliations:** 1 Laboratory of Enzyme Engineering and Microbiology, University of Sfax, National School of Engineering, PO Box 1173-3038, Sfax, Tunisia; E-Mail: islem.younes@gmail.com; 2 Biomaterials Applications, 6 rue Lesdiguières, Grenoble 38000, France

**Keywords:** chitin, chitosan, chemical and enzymatic deproteinization, demineralization, characterization, deacetylation, biological activities, biomedical applications

## Abstract

This review describes the most common methods for recovery of chitin from marine organisms. In depth, both enzymatic and chemical treatments for the step of deproteinization are compared, as well as different conditions for demineralization. The conditions of chitosan preparation are also discussed, since they significantly impact the synthesis of chitosan with varying degree of acetylation (DA) and molecular weight (MW). In addition, the main characterization techniques applied for chitin and chitosan are recalled, pointing out the role of their solubility in relation with the chemical structure (mainly the acetyl group distribution along the backbone). Biological activities are also presented, such as: antibacterial, antifungal, antitumor and antioxidant. Interestingly, the relationship between chemical structure and biological activity is demonstrated for chitosan molecules with different DA and MW and homogeneous distribution of acetyl groups for the first time. In the end, several selected pharmaceutical and biomedical applications are presented, in which chitin and chitosan are recognized as new biomaterials taking advantage of their biocompatibility and biodegradability.

## 1. Introduction

**Figure 1:**
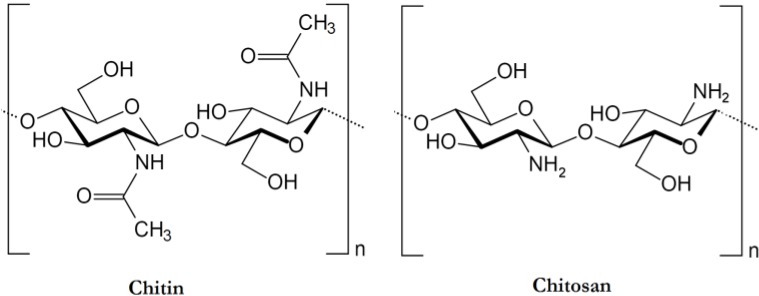
Chemical structure of chitin and chitosan

Chitin or poly (β-(1→4)-*N*-acetyl-d-glucosamine) is a natural polysaccharide of major importance, first identified in 1884 (Figure 1). This biopolymer is synthesized by enormous number of living organisms [1] and it belongs to the most abundant natural polymers, after cellulose. In the native state, chitin occurs as ordered crystalline microfibrils which form structural components in the exoskeleton of arthropods or in the cell walls of fungi and yeast. So far, the main commercial sources of chitin are crab and shrimp shells. In industrial processing, chitin is extracted by acid treatment to dissolve the calcium carbonate followed by alkaline solution to dissolve proteins. In addition, a decolorization step is often added in order to remove pigments and obtain a colorless pure chitin. All those treatments must be adapted to chitin source, owing to differences in the ultrastructure of the initial material (the extraction and pre-treatments of chitin will be described later), to produce first a high quality chitin, and then chitosan (after partial deacetylation). Chitin is infusible and sparingly soluble during transformation into different conformations. The question of its solubility is a major problem in the development of both processing and use of chitin as well as its characterization.

Chitin has more applications while transforming to chitosan (by partial deacetylation under alkaline conditions) [2,3,4]. Chitosan is a random copolymer with a molar fraction DA (degree of acetylation) of β-(1→4)-*N*-acetyl-d-glucosamine (Figure 1) and a fraction (1-DA) of β-(1→4)-d-glucosamine (Figure 1). The degree of acetylation of chitosan is characterized by the molar fraction of *N*-acetylated units (DA) or as a percentage of acetylation (DA%).

This review aims to present the state-of-the-art knowledge on the morphology of chitin and chitosan, the main techniques applied to chitin isolation and chitosan production. Then, the best methods for characterization in solution or solid state are also indicated. It is pointed out that for biomedical products, chitin and chitosan need to be highly purified, since residual proteins and pigments can cause side effects. Finally, the main biological properties will be analyzed in relation with the chemical structure (degree of acetylation and molecular weight of chitosan).

Concerning applications of chitin and chitosan, several examples used for drug release, wound dressing or biofilms are described. It is important to recall that chitin is a natural polymer as well as biocompatible and biodegradable in the body, thus widely used for biomedical and pharmaceutical applications. Additionally, good film forming properties are valuable for wound dressing, artificial skin or packaging.

## 2. Chitin Preparation and Characterization

### 2.1. Morphology of Chitin

Depending on its source, chitin occurs as two allomorphs, namely the α and β forms [5,6], which can be differentiated by infrared and solid-state NMR spectroscopies together with X-ray diffraction. In the solid state, chitin chains are assembled by the H-bonds network which controls the solubility, swelling and reactivity.

α-Chitin isomorph is by far the most abundant; it occurs in fungal and yeast cell walls, in krill, lobster and crab tendons and in shrimp shells, as well as in insect cuticle. In addition to the native chitin, α-chitin is systematically formed by: recrystallization from chitin solution [7,8], by *in vitro* biosynthesis [9,10] or enzymatic polymerization [11] due to high thermodynamical stability of this isomorph.

The rarer β-chitin is found in association with proteins in squid pens [5,12] and in the tubes synthesized by pogonophoran and vestimetiferan worms [13,14]. The crystallographic parameters of the two isomorphs allow us to conclude that there are two antiparallel molecules per unit cell in α-chitin but only one in β-chitin in a parallel arrangement. In these two structures, the chains are organized in sheets and held by intra-sheet hydrogen bonds. In addition, in α-chitin, inter-sheet hydrogen bonds prevent diffusion of small molecules into the crystalline phase. No inter-sheet hydrogen bonds are found in the crystal structure of β-chitin. This may explain its swelling in the presence of polar guest molecules (ranging from water to alcohol and amines) which penetrate the crystal lattice without disturbing the sheet organization and the crystallinity of the sample. The removal of the guest molecule allows us to revert to the original state of anhydrous β-chitin. The reactivity of β-chitin isomorph is larger than the α-isomorph, which is important for enzymatic and chemical transformations of chitin [15]. To conclude, both α and β forms are insoluble in all the common solvents. This insolubility is a major problem in the view of the development of processing and applications of chitin.

### 2.2. Chitin Extraction

The main sources of raw material for the production of chitin are cuticles of various crustaceans, principally crabs and shrimps. In crustaceans or more specifically shellfish, chitin is found as a constituent of a complex network with proteins onto which calcium carbonate deposits to form the rigid shell. The interaction between chitin and protein is very intimate and there is also a small fraction of protein involved in a polysaccharide-protein complex [16]. Thus, chitin isolation from shellfish requires the removal of the two major constituents of the shell, proteins by deproteinization and inorganic calcium carbonate by demineralization, together with small amounts of pigments and lipids that are generally removed during the two previous steps. In some cases, an additional step of decolorization is applied to remove residual pigments. Many methods have been proposed and used over the years to prepare pure chitin; however, no standard method has been adopted. Both deproteinization and demineralization could be carried out using chemical or enzymatic treatments. The order of two steps mentioned before may be reversed with some benefit, especially when enzymatic treatment is considered. Microbial fermentation is also employed; in that case deproteinization and demineralization steps are processed simultaneously.

Regardless to the selected treatment, the isolation of chitin begins with the selection of shells. For example, for lobsters and crabs, the selection has important bearing on the subsequent quality of the final isolated material. Ideally, shells of the same size and species are chosen. In the case of shrimps, the wall of shell is thinner, thus the chitin isolation is easier than from other types of shells. The selected shells are then cleaned, dried and ground into small shell pieces.

#### 2.2.1. Chemical Extraction

##### 2.2.1.1. Chemical Deproteinization

The deproteinization step is difficulty due to disruption of chemical bonds between chitin and proteins. This is performed heterogeneously using chemicals which also depolymerize the biopolymer. The complete removal of protein is especially important for biomedical applications, as a percentage of the human population is allergic to shellfish, the primary culprit being the protein component.

Chemical methods were the first approach used in deproteinization. A wide range of chemicals have been tested as deproteinization reagents including NaOH, Na_2_CO_3_, NaHCO_3_, KOH, K_2_CO_3_, Ca(OH)_2_, Na_2_SO_3_, NaHSO_3_, CaHSO_3_, Na_3_PO_4_ and Na_2_S. Reactions conditions vary considerably in each study. NaOH is the preferential reagent and it is applied at concentration ranging from 0.125 to 5.0 M, at varying temperature (up to 160 °C) and treatment duration (from few minutes up to few days). In addition of deproteinization, the use of NaOH invariably results in partial deacetylation of chitin and hydrolysis of the biopolymer lowering its molecular weight.

##### 2.2.1.2. Chemical Demineralization

Demineralization consists in the removal of minerals, primarily calcium carbonate. Demineralization is generally performed by acid treatment using HCl, HNO_3_, H_2_SO_4_, CH_3_COOH and HCOOH [17,18]. Among these acids, the preferential reagent is dilute hydrochloric acid. Demineralization is easily achieved because it involves the decomposition of calcium carbonate into the water-soluble calcium salts with the release of carbon dioxide as shown in the following equation:

2 HCl + CaCO_3_ → CaCl_2_ + H_2_O + CO_2_ ↑

Most of the other minerals present in the shellfish cuticle react similarly and give soluble salts in presence of acid. Then, salts can be easily separated by filtration of the chitin solid phase followed by washing using deionized water.

Demineralization treatments are often empirical and vary with the mineralization degree of each shell, extraction time, temperature, particle size, acid concentration and solute/solvent ratio. The latter depends on the acid concentration, since it needs two molecules of HCl to convert one molecule of calcium carbonate into calcium chloride. In order to have a complete reaction, acid intake should be equal to the stoichiometric amount of minerals, or even greater. [19,20]. Since, it is difficult to remove all minerals (due to the heterogeneity of the solid), larger volume or more concentrated acid solution is used. Demineralization can be followed by acidimetric titration: the evolution of pH towards neutrality corresponds to acid consumption but the persistence of acidity in the medium indicates the end of the reaction [21].

Several demineralization treatments were previously used, involving various reaction conditions. Conventionally, demineralization is accomplished using dilute hydrochloric acid at different concentrations (up to 10% w/v) at room temperature, during different incubations time (Table 1). Among such methods are those of Muzzarelli *et al.* [22], Hackman [23,24], Anderson *et al.* [25] (Table 1).

Exceptions to the above are seen in the methods of Horowitz *et al.* [26] and Synowiecki *et al.* [27] where demineralization was accomplished with 90% formic acid and 22% HCl, respectively, at room temperature. Most of the aforementioned methods include drastic treatments that may cause modifications, such as depolymerization and deacetylation of native chitin [28]. In order to overcome this problem, other methods have been developed using mild acids (to minimize degradation). For instance, Austin *et al.* [29] used ethylenediaminetetracetic acid (EDTA), Brine and Austin [30] applied acetic acid. Peniston and Johnson [31] studied a sulfurous acid process, *etc.* However, these treatments resulted in chitins with high residual ash content.

Demineralization using HCl usually can be achieved in 2 to 3 h under stirring [19]. However, reaction time varies with preparation methods from 15 min [18] to 48 h as seen in Table 1. Longer demineralization time, even to several days, results in a slight drop in the ash content but also causes polymer degradation [32,33].

Moreover, it was reported that the use of high temperature accelerates the demineralization reaction by promoting the penetration of the solvent into the chitin matrix. Thus, some demineralization reactions were carried out at higher temperature [34]. Furthermore, it was reported that the penetration of solvent into the chitin matrix strongly depends on the particles size. According to Marquis-Duval [35], the decisive factor in the demineralization is related to the contact area between the chitin matrix and the solvent. However, it was reported that high temperatures, longer incubations, high acid concentrations and granulometry affect the final physico-chemical properties of the resulting chitin.

In conclusion, although many experimental conditions can be found in the literature for the removal of minerals, effects on the molecular weight and acetylation degree cannot be avoided. Only Percot *et al.* [18] studied the extraction of chitin from shrimp shells using mild conditions allowing them to obtain chitin with a high DA. The demineralization was performed under the following conditions: at room temperature, in the presence of stoichiometric amount of 0.25 M HCl with regards to the calcium carbonate content, for 15 min incubation time. Deproteinization was later classically processed at 70 °C for 24 h using 1 M NaOH. These conditions well preserve the chitin structure, with a high DA remaining above 95%. Unfortunately, the amount of residual proteins and minerals were not determined and the influence on MW was not studied.

**Table 1:** Comparison of conditions for chitin production according to literature.

Source	Deproteinization				Demineralization			References
	NaOH Concentration *	Temperature (°C)	Number of Baths	Duration (h)	HCl Concentration*	Temperature (°C)	Duration (h)	
12 species of crustaceous and cephalopods	0.3 M	80–85	From 2 to 7 according to the source	1 h for each bath	0.55 M	Room	15 mn to 1 h by bath repeated 2–5 times according to the source	[21]
Shrimp	0.125 M	100	1	0.5	1.25 M	Room	1	[36]
	0.75 M	100	1	-				
Shrimp	1.25 M	100	1	0.5	1.57 M	20–22	1–3	[37]
Crab	0.5 M	65	1	2	1.57 M	Room	5	[22]
Crab	1 M	80	1	3	1 M	Room	12	[38]
Crab	1 M	100	1	36	2 M	Room	48	[32]
Crab	1 M	100	3	72	1 M	Room	-	[23]
Crab	1.25 M	85–90	3	24	1.37 M	Room	24	[39]
Crab/Lobster	2.5 M	Room	3	72	11 M	−20	4	[40]
Krill	0.875 M	90–95	1	2	0.6 M	Room	2	[25]
Lobster	1 M	100	5	12	2 M	Room	5	[24]
Squid	2 M	Room	2	One night	1 M	Room	One night	[41]
	2 M	100		4				
Lobster	10%	100	1	2.5	10% HCl 90% formic	Room	18	[26]
Krill	3.5%	25	1	2	3.5%	20	1.5	[42]
Lobster	5%	80–85	2	0.5	5%	70	4	[43]
Crawfish	3.5%	65	1	2	1 M	Room	0.5	[44]
Crab	1 M	50	1	6	1 M	20	3	[30]
Shrimp	1%	65	1	1	0.5 M	Room	-	[45]
Shrimp	3%	100	1	1	1 M	Room	0.5	[46]
Shrimp	4%	100	1	1	5%	Room	-	[47]

* Reactant concentrations are expressed in molarity or w/v %.

##### 2.2.1.3. Processes Preserving Chitin Structure

To the best of our knowledge, there have been no studies which conduct a production of chitin with the highest DA and MW, free of minerals and proteins.

However, only the partial deacetylation may be controlled (using solid state ^13^C-NMR). Furthermore, the chain degradation can be also evaluated by viscometry but after additional treatment, *i.e.*, solubilization of residual chitin in specific solvent, or after conversion to a soluble product (chitosan). Nevertheless, in the last case, the deacetylation process is usually accompanied by polymer degradation. Thus, to estimate the influence of chitin extraction process, only DA is determined as indication of the degree of the chitin degradation. It may be assumed that the higher the DA is obtained for an extracted chitin, the less the polymer is degraded.

Optimized extraction method of pure chitin production with maximum preservation of its structure (MW, DA) allows us to get chitin corresponding to the native chitin in the cuticle structure. This approach was proposed by Tolaimate *et al.* [21] who used chemical treatments for both demineralization and deproteinization.

In the study of Tolaimate *et al.* [21], a new approach was proposed using successive baths of lower HCl (0.55 M) and NaOH (0.3 M) concentrations. The number of baths for each step was dependent on the tested animal species. This method has proved a good efficacy on the reduction of proteins and minerals as well as preservation of the native chitin form for 12 different species of crustaceous and cephalopods (Table 2). The DA of the prepared chitins, determined with ^13^C-NMR, was varying between 96% and 100% for all the species. For example, for shrimp shells, extracted chitin was 100% acetylated. So far, such high degree of deacetylation has never been mentioned in the literature.

**Table 2:** Comparison of chitin production from different sources according to Tolaimate *et al.* [48].

Source		Number of Deproteinization Baths	Number of Demineralization Baths	DA
		0.3 M; NaOH 80 °C; 1 h	0.55 M HCl; 25 °C; 2 h	
Cirripedia	Anatife	4	2	100
Reptantia Brachyura	Red crab	3	5	97
	Marbled crab	3	3	99
	Spider crab	3	3	96
Reptantia Macrura	Lobster	3	3	-
	Crayfish	7	3	100
	slipper lobster	3	2	-
	Freshwater crayfish	3	2	-
Natantia	Pink shrimp	3	3	100
	Grey Shrimp	2	2	100
Stomatopoda	Squilla	3	3	100
Cephalopoda	Squid	2	2	100

#### 2.2.2. Biological Extraction of Chitin

The extraction by chemical treatments has many drawbacks: (i) it harms the physico-chemical properties of chitin and leads to MW and DA decrease that negatively affects the intrinsic properties of the purified chitin; (ii) it affects wastewater effluent that contains some chemicals (iii) it increases the cost of chitin purification processes. Furthermore, the development of the green extraction techniques based on the concept of ‘Green chemistry’ is gaining greater attention, favoring the application of enzymes and microorganisms for chitin extraction. A comparative study was carried out by Khanafari *et al.* [49] for extraction of chitin from shrimp shells by chemical and biological methods. The results indicated that the biological method (using microorganisms) was better than the chemical one because it preserves the structure of chitin. Bustos and Healy [50] also demonstrated that chitin obtained by the deproteinization of shrimp shells with various proteolytic microorganisms has higher molecular weights in comparison with chemically prepared shellfish chitin. The biological extraction of chitin offers high reproducibility in shorter time, simpler manipulation, smaller solvent consumption and lower energy input. However, the biological method is still limited to laboratory scale studies.

Recently, two reviews have reported the most common biological methods used for chitin extraction [51,52], *i.e*., the use of proteolytic enzymes in order to digest the proteins or a fermentation process using microorganism which allows a digestion of both proteins and minerals.

The use of enzymes in the deproteinization step was first mentioned in the original Rigbv patent from 1934 but there has been a renewed interest in this approach since 1977. This work has led to the lactic acid bacterial fermentation process, studied more extensively later by Guerrero Legarreta *et al*. [53] and Cira *et al*. [54].

##### 2.2.2.1. Enzymatic Deproteinization

Chitin extraction requires the use of proteases. Proteolytic enzymes are mainly derived from plant, microbial and animal sources. Many proteases such as alcalase, pepsin, papain, pancreatine, devolvase and trypsin remove proteins from crustacean shells and minimize the deacetylation and depolymerization during chitin isolation. This treatment may be performed either after, or before demineralization step of the solid material, which modifies the accessibility for the reactants.

Both purified and crude extracted proteases are used in the deproteinization step. However, commercially purified enzymes are expensive in contrast to crude proteases, which are not only cheaper but also more efficient due to the presence of coexisting proteases. Crude proteases are mainly derived from bacteria and fish viscera, bacterial proteases being the most common. Marine animals possess the same functional classes of enzymes, which are present in animal tissues and may be recovered in both active and stable forms for commercial use. In several of the major fish producing countries, the by-products represent about 50% of the seafood harvest [55]. These materials are largely underutilized and discarded as waste. Thus, application of these crude enzymes in the chitin extraction process could be interesting in decreasing the costs of this process as well as in preserving the environment.

It must be noted that the efficiency of enzymatic methods is inferior to chemical methods with approximately 5%–10% residual protein typically still associated with the isolated chitin. The final isolated chitin could be then treated with an additional NaOH treatment (under milder conditions and for a shorter time) to increase its purity and preserve the structure of chitin.

Many reports have demonstrated the application of bacterial proteases in deproteinization step. For example, Synowiecki and Al-Khateeb [56] applied enzymatic deproteinization on previously demineralized shrimp waste in order to produce chitin and a nutritionally valuable protein hydrolyzate. Alcalase 2.4 L (Novo Nordisk A/S), a serine endopeptidase obtained from *Bacillus licheniformis*, was used. This enzyme was selected due to its specificity for terminal hydrophobic amino acids, which generally leads to the production of non-bitter hydrolyzate and allows an easy control of the hydrolysis degree. The obtained hydrolyzate is a good source of essential amino acids in food applications. However, the effectiveness of deproteinization was limited by the presence of residual small peptides and amino acids attached to chitin molecules which persist after enzymatic hydrolysis. This method allows the isolation of chitin containing about 4% of protein impurities. Such purity is sufficient for many non-medical applications of chitin. Gilberg and Stenberg [57] also used alcalase 2.4 L for chitin, protein hydrolyzate and asthaxanthin recovery.

Manni *et al.* [58] compared the isolation of chitin from shrimp waste using *Bacillus cereus* SV1 crude alkaline proteases to the use of 1.25 M NaOH. Shrimp shells were demineralized after deproteinization using dilute HCl treatment. The residual protein content was significantly higher in the chitin isolated with the enzymatic deproteinization than that obtained with alkali treatment (10% compared to 6%).

In another study, enzymatic deproteinization was optimized by Younes *et al.* [59] before demineralization. In this study many microbial proteases were compared on the basis of their efficiency in shrimp shells deproteinization. Six alkaline crude microbial proteases from *Bacillus mojavensis* A21, *Bacillus subtilis* A26, *B. licheniformis* NH1, *B. licheniformis* MP1, *Vibrio metschnikovii* J1 and *Aspergillus clavatus* ES1, were used. The highest deproteinization degree was obtained with *B. mojavensis* A21 proteases, being at about 76%. Then, the effect of reaction conditions, *i.e*., mainly enzyme/substrate ratio, temperature and incubation time, on the deproteinization degree were optimized using response surface methodology to reach 88% deproteinization under the optimized conditions.

Recently many fish and marine invertebrate alkaline crude proteases have been applied for shrimp shell deproteinization. Mukhin and Novikov [60] studied the possibility of using crustacean waste both as a substrate and as a source of proteases. The shell proteins were degraded with crude proteases isolated from the hepatopancreas of crab. The objective was to optimize the protein hydrolyzate yield. However, even under the best conditions, *i.e*., temperature = 50 °C, time = 12 h, pH = 8.4, Enzyme/Substrate ratio of 6 g/kg, the degree of hydrolysis was never higher than 80%.

Younes *et al*. [61] used alkaline proteases from the red scorpionfish *Scorpaena scrofa* for shrimp waste deproteinized up to 85%. Activities of these crude alkaline proteases are probably related to the fish feeding mainly on crustaceans and mollusks inducing the nature and the specificity of its enzymes.

By contrast, when extraction is carried out by chemical process, the order of two steps (deproteinization and demineralization) does not have significant effect on the quality and the yield of the final chitin [62]. However, if enzymatic deproteinization is applied, the minerals presented in the cuticles may decrease the accessibility of the proteases and affect shrimp shells deproteinization efficiency. Thus, demineralization should be performed firstly.

##### 2.2.2.2. Fermentation

The cost of using enzymes can be decreased by performing deproteinization by fermentation process, which can be achieved by endogeneous microorganisms (called auto-fermentation) or by adding selected strains of microorganisms. This latter can be achieved by single-stage fermentation, two-stage fermentation, co-fermentation or successive fermentation.

Many microorganism species were proposed for crustacean shells fermentation as summarized by Arbia *et al.* [51]. Fermentation methods could be separated into two major categories: lactic acid fermentation and non-lactic acid fermentation.

###### (a) Lactic Acid Fermentation

Fermentation of crustacean shells can be performed by selected *Lactobacillus* sp. strain as inoculum which produces lactic acid and proteases. Lactic acid is obtained by conversion of glucose resulting in lower pH condition of silage suppressing the growth of spoilage microorganisms. Lactic acid reacts with the calcium carbonate, leading to the formation of a precipitate of calcium lactate separated from lighter shells which are recovered and rinsed with water. This process may be realized either on purified crustaceous shells, or on complete shrimp waste (including heads and viscera). Thus, deproteinization and simultaneous liquefaction of the proteins could occurred by action of proteases produced by added strains, or by gut bacteria present in the intestinal system of the treated shrimps, or by proteases present in the biowaste itself. The efficiency of lactic acid fermentation depends on many factors, mainly the species and quantity of inoculums, carbon source and its concentration, initial pH and pH evolution during fermentation, temperature and the duration of fermentation [63,64,65].

For example, Choorit *et al.* [66] used response surface methodology to optimize demineralization efficiency in fermented shrimp shells. Following variables were tested: sucrose concentration, initial pH value and soaking time, using *Pediococcus* sp. L1/2. Results showed an increase in demineralization degree (caused by higher sucrose concentration and soaking time) as well as an important effect of the initial pH. Demineralization degree reached at about 83% at pH 7, compared to 68% at pH 6 (sucrose concentration 50 g/L and soaking time 72h).

Otherwise, Rao *et al*. [64] studied the effect of different fermentation parameters (initial pH, initial glucose concentration and inoculation with different quantities of *Lactobacillus*) on deproteinization and demineralization degrees. Combined treatment with *Lactobacillus* and reduction of initial waste pH by addition of acetic acid produced lower deproteinization and higher demineralization degrees than treatment with *Lactobacillus* or acid individually. In addition, inoculation with *Lactobacillus* resulted in a high-quality protein liquor output, whereas autofermented waste (due to the presence of shrimp microflora) gave a strong stinky protein fraction. In the fermentation with lactic acid bacteria, the demineralization efficiency and the quality of the derived product are high, and the addition of commercial proteases may even increase deproteinization.

###### (b) Non Lactic-Acid Fermentation

In non-lactic acid fermentation, both bacteria and fungi were used for crustacean shells fermentation, for example: *Bacillus* sp. [67,68,69], *Pseudomonas* sp. [65,70,71] and *Aspergillus* sp. [72].

Ghorbel-Bellaaj *et al*. [69] evaluated six proteolytic *Bacillus* strains on the fermentation of shrimp waste: *Bacillus pumilus* A1, *B. mojavensis* A21, *B. licheniformis* RP1, *B. cereus* SV1, *B. amyloliquefaciens* An6 and *B. subtilis* A26. Results showed that all the *Bacillus* strains were able to deproteinize shrimp waste. The highest deproteinization degree was obtained using *B. cereus* SV1. These authors had also tested the role of additional amount of glucose on fermentation and they concluded that glucose had no significant effect on deproteinization degree and improved demineralization.

Sini *et al*. [68] had studied fermentation of shrimp shells in jaggery broth using *B. subtilis.* About 84% of the proteins and 72% minerals were removed; after this step the residue was treated with 0.8 N HCl and 0.6 N NaOH to reduce residual proteins and minerals to satisfactory level at about 0.8% proteins and 0.8% minerals.

Many factors have been reported to influence the fermentation process and consequently deproteinization and demineralization efficiencies [65,66,73]. Ghorbel-Bellaaj *et al*. [70] used Plackett-Burman factorial design to screen the main factors influencing fermentation efficiency using *P. aeruginosa* A2. This method is thus quite useful in preliminary studies, in which the main objective is to select variables that can be fixed or eliminated in further optimization process. Only four variables were reported to be effective on deproteinization and demineralization degrees: shrimp shell concentration, glucose concentration, inocculum size and incubation time. Under these conditions: initial medium pH, temperature, speed of agitation and volume of culture no effect on fermentation efficiency was observed. Then, from response surface methodology, under optimal conditions for fermented shrimp shells, maximum demineralization was 96%, and deproteinization was 89% [70].

Proteolytic enzymes released from fungus *A. niger* were also tested for their deproteinization and demineralization efficiency of crustacean shells. Teng *et al*. [74] evaluated concurrent production of chitin from shrimp shells and fungi in a one-pot fermentation process where proteases from the fungi hydrolyze proteins into amino acids that in turn act as a nitrogen source for fungal growth. Results showed that residual proteins in the isolated shrimp chitin were below 5%. The protein content in the fungal chitin was higher (10%–15%). They concluded that fungi and shrimp shells supplementation with glucose in a single reactor led to release of protease by the fungi and enhance the deproteinization of shrimp shells. The hydrolyzed proteins in turn were utilized for fungal growth, leading to lower pH of the medium and further demineralization of the shrimp shells.

The various biological methods of chitin extraction by microorganisms are simple, more productive and environmentally friendly when compared to chemical processes. However, microbial fermentation has its drawbacks such as: longer processing time compared to chemical methods, poorer accessibility of proteases (caused by the presence of minerals which lead to high residual proteins). Nevertheless, deproteinization rate could be ameliorated depending on the end use requirements in particular for biomedical applications. This could be achieved by using simultaneous or successive processes such as two-step fermentations or co-fermentation of microorganisms. In order to obtain highly purified chitin, biotechnological process must be completed by further mild chemical treatment to remove the residual protein and minerals.

Recently, Gortani and Hours [52] concluded that a cost-effective, fast, and easily controllable industrial process for producing chitin of high MW and DA still requires further development and optimization of the extraction process, such as: minimization of chitin degradation and decrease the impurity levels to a satisfactory level highly desirable for specific applications.

### 2.3. Chitin Characterization and Solubility

**Figure 2:**
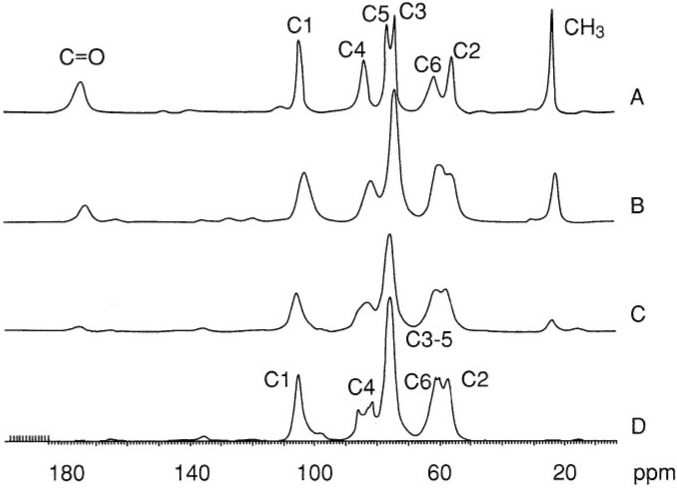
^13^C NMR spectra for (**A**) chitin and chitosans (**B**) obtained by homogeneous reacetylation DA = 0.60; (**C**) commercial chitosan from Pronova DA = 0.2; (**D**) fully deacetylated chitin. Reproduced with permission from [90]. Copyright 2000 American Chemical Society.

In the solid state, the chains are parallel in β-chitin and antiparallel in α-chitin [75]. Their crystalline structures were reviewed in different papers using X-ray diffraction method [76,77,78,79], IR spectroscopy [80,81,82,83,84,85,86], and NMR [87,88,89,90]. Solid state ^13^C-NMR is a commonly used technique for differentiation of the two isomorphs [88], for determination of the deacetylation degree of chitin (DD) and for control of purification conditions. Figure 2 shows typical spectra for α-chitin and chitosans with different acetylation degrees [90].

Unfortunately, physical properties of chitin in solution cannot be analyzed correctly due to poor data caused mainly by difficulties in dissolution of this polymer. Dissolution is desired to estimate the molecular weight but also to process chitin (chitin cannot be processed in molten state). Presence of aggregates in solution precludes light scattering measurements and overestimates the molecular weights. Therefore, the most applicable technique here is viscometry where the Mark-Houwink parameters are known under defined thermodynamic conditions used (solvent, temperature). One of the mostly known system is based on complex formation between chitin and LiCl (at 5 wt% in the solvent DMAC) in dimethylacetamide solvent. Experimental values of parameters *K* and *a* relating intrinsic viscosity [η] and molecular weight M for chitin in this solvent are estimated from well-known Mark-Houwink equation according to:

[η] (mL/g) = KM^a^ (1)

with K = 7.6 × 10^−3^ , a = 0.95 at 30 °C [91] and K = 2.4 × 10^−1^, a = 0.69 at 25 °C [92].

A review on chitin and chitosan including the question of their solubility was recently published in relation to fiber processing [93]. It has been demonstrated that chitin is able to be processed from solutions. Additionally, chitin, like other polysaccharides derived from cellulose, has good film-forming properties and good stability promoted by the establishment of a hydrogen bond network between extended chains. Chitin gives original properties to the new materials due to its biocompatibility, biodegradability and non-toxicity, with antimicrobial activity and low immunogenicity.

## 3. Chitosan Preparation and Characterization

### 3.1. Chitosan Preparation

The term chitosan usually refers to a family of polymers obtained after chitin deacetylation to varying degrees. In fact, the acetylation degree, which reflects the balance between the two types of residues (Figure 1), differentiates chitin from chitosan. When the DA (expressed as molar percentage) is lower than 50 mol%, the product is named chitosan and becomes soluble in acidic aqueous solutions [94]. During deacetylation, acetyl groups are removed but also depolymerization reaction occurs, indicated by changes in MW of chitosan.

Chitin can be converted to chitosan by enzymatic preparations [95,96,97,98] or chemical process [99,100]. Chemical methods are used extensively for commercial purpose of chitosan preparation because of their low cost and suitability to mass production [100].

#### 3.1.1. Chemical Deacetylation

From a chemical point of view, either acids or alkalis can be used to deacetylate chitin. However, glycosidic bonds are very susceptible to acid; therefore, alkali deacetylation is used more frequently [100,101].

The *N*-deacetylation of chitin is either performed heterogeneously [102], or homogeneously [103]. Commonly, in the heterogeneous method, chitin is treated with a hot concentrated solution of NaOH during few hours, and chitosan is produced as an insoluble residue deacetylated up to ∼85%–99%. According to the homogeneous method, alkali chitin is prepared after dispersion of chitin in concentrated NaOH (30 g NaOH/45 g H_2_O/ 3 g Chitin) at 25 °C for 3 h or more, followed by dissolution in crushed ice around 0 °C. This method results in a soluble chitosan with an average degree of acetylation of 48%–55% [99]. This process produces deacetylation with acetyl groups uniformly distributed along the chains, for example chitosan with DA = 10% after 580 h at 25 °C [103].

Rinaudo and Domard [104] reported that the solubility of chitosan can be characterized not only by the fraction of 2-acetamido-2-deoxy-d-glucose units in the molecule but also by the *N*-acetyl group distribution. Aiba [105] showed that deacetylation reaction performed under heterogeneous conditions gives an irregular distribution of *N*-acetyl-d-glucosamine and d-glucosamine residues with some blockwise acetyl group distribution along polymeric chains. Thus, solubility and degree of aggregation of chitosan can vary in aqueous solutions leading to changes in their average characteristics. For instance, physico-chemical properties of such chitosans may differ from those of randomly acetylated chitosans obtained under homogeneous conditions.

Furthermore, variations in chitosan preparation may also result in changes of: DA, distribution of acetyl groups along the chains, MW and viscosity in solution [106,107].

In fact, many parameters in the deacetylation reaction can impact the characteristics of the final chitosan [108]. For instance, Rege and Block [109] had investigated the effect of temperature, processing time and mechanical shear on chitosan characteristics, and found that temperature and processing time have a significant effect on DA and MW. Tolaimate *et al.* [110] reported that chitosan DA is greatly affected by temperature and repetition of alkaline steps. Wu and Bough [45] studied the effects of time and NaOH concentration. Tsaih and Chen [111] also examined the effect of time reaction and temperature. All these studies were conducted using a classical one-variable-at-a-time experimentation. These reports indicate that MW and DA of chitosan are mainly affected by NaOH concentration, reaction time, temperature and repetition of alkaline steps. Additional factors such as reaction reagent, atmosphere, particle size, chitin and solvent ratio, and source of raw material were also tested in others studies [100,102,110,112].

Weska *et al.* [113] attempted to optimize chitin deacetylation by response surface methodology (controlling MW and/or DA) using temperature and reaction time variables. Hwang *et al.* [114] studied the effect of temperature, time and NaOH concentration on the deacetylation. Chang *et al.* [102] reported the influence of NaOH concentration, temperature and solution/chitin ratio and found that chitosan DA was decreasing with increase of temperature and NaOH concentration. Other parameters, such as: the use of alkali successive baths, atmospheric conditions and presence of different additives could influence deacetylation but were not considered previously in optimization studies.

Deacetylation was investigated using seven factors: the alkali reagent, its concentration, temperature, reaction time, the use of successive baths, atmospheric conditions and the use of sodium borohydride, a reducing agent [115]. For that purpose, a fractional factorial design was applied and a mathematical model was established to allow optimizing experimental conditions for chitosan of desired DA. Results clearly revealed a significant effect of temperature and the alkali reagent nature (NaOH treatment is much more efficient than KOH). It has been found that DA is significantly affected by the use of successive baths, reaction time and alkali concentration. By contrast, the atmospheric conditions (nitrogen or air) and the use of a reducing agent (NaBH_4_) do not have significant effect on the DA of chitosan but MW of chitosan was higher under atmospheric nitrogen and addition of sodium borohydride which prevents polymer degradation. These results are in agreement with previous ones obtained with thiophenol and NaBH_4_ used as oxygen scavenger and reducing agent, respectively [116].

#### 3.1.2. Enzymatic Deacetylation

Chemical deacetylation has also disadvantages: energy consumption; waste of concentrated alkaline solution, thus an increase of environmental pollution, broad and heterogeneous range of soluble and insoluble products.

In order to overcome these drawbacks in the chitosan preparation, an alternative enzymatic method exploiting chitin deacetylases has been explored. The use of chitin deacetylases for the conversion of chitin to chitosan, in contrast to the currently used chemical procedure, offers the possibility of a controlled, non-degradable process, resulting in the production of novel, well-defined chitosan [117]. This method is specially used to prepare chitosan oligomers.

Chitin deacetylase (EC 3.5.1.41) catalyzes the hydrolysis of *N*-acetamido bonds in chitin to produce chitosan. The presence of this enzyme activity has been reported in several fungi [118,119,120,121,122,123] and insect species [124]. The mostly well-studied enzymes are those extracted from the fungi *Mucor rouxii* [95,118,119], *Absidia coerulea* [120], *Aspergillus nidulans* [121] and two strains of *Colletotrichum lindemuthianum* [122,123]. All the enzymes are glycoproteins and are secreted either into the periplasmic region or into the culture medium. Furthermore, all enzymes exhibit a remarkable thermal stability at their optimal temperature (50 °C), and exhibit a very strong specificity for β-(1,4)-linked *N*-acetyl-d-glucosamine polymers. However, the enzymes vary significantly in their MW and carbohydrate content and display a wide range of pH optima. It is interesting to notice that chitin deacetylases, produced by *C. lindemuthianum* and *A. nidulans,* are not inhibited by acetate (a product of the deacetylation) which make them suitable for potential biotechnological applications [121,122,123].

The efficiency of chitin deacetylase, isolated from the fungus *M. rouxii*, on the chitosan preparation was tested using chitin as a substrate (both in its crystalline and amorphous morphology) [125]. Deacetylation degrees remain very low (<10%) indicating that the enzyme is not really effective on insoluble chitins. Similar results were also obtained using chitin deacetylases isolated from other sources [120,122,123]. Thus, pretreatment of chitin substrates before enzyme addition seems to be necessary in order to improve the accessibility of the acetyl groups to the enzyme and therefore to enhance the deacetylation yield. For that purpose, experiments have been performed in homogeneous conditions using chitin deacetylase from *M. rouxii* with partially deacetylated water-soluble chitosans [126]. In selected conditions, the enzyme is able to deacetylate chitosan up to 97% (deacetylation from an initial chitosan with DA = 0.32 and a number-average degree of polymerization of 30) [126].

These findings suggest that the development of a controllable process using the enzymatic deacetylation on chitinous substrates is an attractive alternative process that can result in the preparation of novel chitosan polymers and more interestingly oligomers.

### 3.2. Chitosan Characterization and Solubility

Chitosan obtained from partial deacetylation of chitin becomes soluble in aqueous acidic medium when the average degree of acetylation DA is lower than 0.5. In fact, this limit depends on the distribution of acetyl groups along the chains. At this stage, it is possible to obtain a complete characterization of the polymer but it may differ from the starting material, specially its molecular weight is reduced during deacetylation in strong alkaline medium, as mentioned previously.

The physical properties of chitosan in solution depend strongly on DA and on the acetyl group distribution along the chains. Block-wise distribution of acetyl groups, caused by heterogeneous deacetylation performed on solid state chitin, causes chain association even in dilute solutions and formation of aggregates as well as difficulties in molecular weight determination [127,128]. In addition, fully deacetylated chitosan may be reacetylated in homogeneous phase [129] in order to get samples soluble in acidic conditions up to DA∼0.6 in relation with a random distribution of the acetyl groups. Under these conditions, the existence of free -NH_2_ groups, available on C-2 position of d-glucosamine units along the chains, allows to perform specific reactions in homogeneous conditions [130,131,132,133,134,135,136].

The first step in chitosan characterization is the determination of their molecular weights (after dissolution), then DA and eventually the distribution of acetyl group along the chain (by NMR). Additionally, different solvents based on acetic acid have been proposed, for instance: 0.3 M acetic acid added with sodium acetate (up to 0.1 or 0.2 M) in aqueous solution. The presence of an external salt is needed to screen the long-range electrostatic repulsions between the charged chains. Chitosan is also soluble in acetic acid or hydrochloric acid at pH lower than 6 (its intrinsic pK being around 6.5) [1].

**Figure 3:**
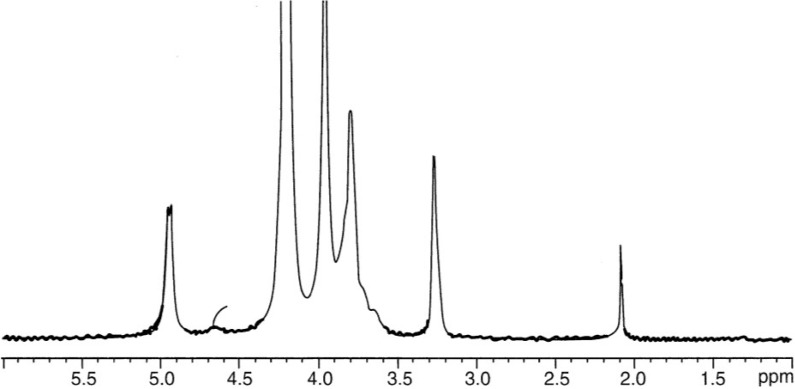
^1^H-NMR spectrum of chitosan with DA~0.06 in D_2_O at pH~4, T = 85 °C and polymer concentration 5 g/L. Signals at 4.9 ppm is for H-1 of d-glucosamine unit, at 4.7 ppm is for H-1 of *N*-acetyl-d-glucosamine, at 3.2 ppm is for H-2 and at 2.1 ppm is for -CH_3_ of the acetyl group allowing to get DA.

The determination of average DA for chitosan may be performed by different techniques: infrared spectroscopy [86], elementary analysis, and potentiometric titration, but ^1^H liquid state [89] and solid state ^13^C-NMR [90,137,138] are preferred. Infrared spectroscopy has to be used carefully because the result interpretation is related to the difficulty in adopting a convenient base line. This problem was also discussed previously for samples with different DAs [86]. At present, ^1^H NMR seems to be the most convenient technique to get the correct DA for soluble samples. An example is given in Figure 3. Additionally, ^13^C NMR is also convenient for DA determination in the case of pure chitin up to fully deacetylated chitosan with a good agreement between measurements in solid state and liquid phase (Figure 2) [90].

It is important to recall that high field ^13^C-NMR spectroscopy is important to establish the distribution of acetyl groups along the chitosan chains [139,140].

Furthermore, the molecular weight distribution and the average molecular weight as well as the intrinsic viscosity also play a significant role. However, chitosan solution must be free of aggregates, thus the solvent for chitosan must be chosen carefully. The viscometric-average molecular weight has been usually calculated from intrinsic viscosity (lower impact of small fractions of aggregates) using the Mark-Houwink relationship [1]. In order to determine *K* and *a* parameters, an absolute MW has to be calculated using light scattering technique; nevertheless, the obtained value is usually overestimated due to high sensitivity to aggregate formation [127,128]. These artifacts may be omitted for instance by the use of 0.3M acetic acid/0.2M sodium acetate (pH = 4.5) solvent which does not form aggregates in this mixture [141]. Under these conditions, the absolute M values were obtained from steric exclusion chromatography (SEC) equipped with viscometer and light scattering detector on line allowing to determine the Mark-Houwink parameters without fractionation and also to obtain the relation between the radius of gyration and the molecular weight [142]. The *K* (mL/g) and *a* parameters at 25 °C are 7.9 × 10^−2^ and 0.796 respectively.

The relatively high values obtained for the parameter *a* are in agreement with the semi-rigid character of this polysaccharide which controls their dimensions, hydrodynamic volume and viscometric contribution. The stiffness is related to the persistence length (*L_t_*) of the chain: chitosan in acid medium behaves like a polyelectrolyte, so the actual total persistence length *L_t_* at a given ionic concentration is equal to the intrinsic contribution *L_p_* and the electrostatic contribution *L_e_*, calculated following the Odijk treatment [143]. The conformational analysis of chitin with different degrees of deacetylation confirms that chitin and chitosan are semi-rigid polymers characterized by a persistence length which depends moderately on the degree of acetylation of the molecule. From this analysis, chitosan, free of acetyl groups, has an intrinsic persistence length *L_p_* of 9 nm in salt excess [144]. *L_p_* increases when DA increases up to 12.5 nm for DA = 0.6, remaining constant up to pure chitin at 25 °C. These predictions are in agreement with the experimental values obtained by SEC [142].

### 3.3. Processing and Main Properties of Chitosan-Based Materials

Solutions of chitosan prepared in acidic medium are processed to the needed conformation (casted for a film, spun for fibers, freeze dried for sponges, *etc.*), immersed in an alkaline solution (in which they precipitate), washed and dried. The processing of chitosan is easier than that of chitin but the stability of the materials is lower due to the larger hydrophilic character and especially the pH sensitivity. For better stability, chitosan may be crosslinked using reagents such as epichlorohydrin, diisocyanate, 1,4-butanediol diglycidyl ether, or glutaraldehyde [145,146,147]. Many chitosan hydrogels were obtained by treatment with multivalent anions as oxalic acid [148,149] or citric acid [150,151,152] or tripolyphosphate [153]. Blends and composites are sometimes produced taking advantage of the polycationic properties of chitosan in acidic conditions.

In fact, chitosan being a polyelectrolyte is able to form interesting electrostatic complexes (hydrogels) with oppositely charged macromolecules. The properties of these complex materials depend on the polymer concentration, temperature, pH and ionic concentration. Electrostatic polyelectrolyte complexes (PEC) are mentioned in the literature involving chitosan complexed with synthetic or natural polymers [4]. Electrostatic interactions between chitosan and lipidic vesicles are also important in the biological and pharmaceutical fields due to bioadhesive and permeabilizer roles of chitosan [154,155,156]. Coating of liposomes with chitosan also increases their biocompatibility, and stabilizes the composite membrane against pH as well as ionic concentration [155].

Nowadays, these electrostatic interactions are applied for preparation of layer-by-layer polyelectrolyte capsules or films based on charged biocompatible polysaccharides or chitosan/synthetic polyelectrolytes [157,158,159]. Core-shell phospholipid nanoparticles were stabilized via layer-by-layer self-assembly of anionic alginate and cationic chitosan and were proposed for protein release [157].

Chitosan and alginate electrostatic complexes have been mostly used so far for biological applications [160,161,162]. Complexes formed between DNA or RNA and chitosan (oligomers or polymers) are actually under further investigation in many laboratories; the charge density and DA of chitosan are essential for the complex stability [163,164,165,166,167].

## 4. Relation between Chemical Structure and Biological Activities

Because chitosan and derivatives possess many beneficially properties such as biocompatibility, biodegradability, safety and also interesting biological activities, much attention has been paid to their applications especially in biomedical, food, biotechnology and pharmaceutical fields [168,169]. Among their attractive biological activities, antimicrobial, antioxidant and antitumor activities will be discussed in detail below. These properties are specially recognized in the field of food preservation and packaging to avoid the use of chemical preservatives and to produce edible antimicrobial films due to the good film forming properties of chitosan. Chitosan, as a polymeric ingredient with a good antimicrobial and antioxidant properties, does not migrate easily out of the protecting film and has better barrier properties. This was previously demonstrated in many studies, such as those of Friedman *et al.* [170], Kardas *et al.* [171] and Alishahi and Aider [172].

Friedman *et al.* [170] studied the antimicrobial activities of chitosan in solution, powders and edible films and coating against foodborne pathogens, spoilage bacteria, and pathogenic viruses and fungi in several food categories. These include fruit juices, eggs and dairy, cereal, meat and seafood products. They suggest that low-molecular-weight chitosans at a pH below 6.0 presents optimal conditions for achieving desirable antimicrobial and antioxidative-preservative effects in liquid and solid foods.

The use of chitosan and derivatives in the food industry was also described in the review of Kardas *et al.* [171]. They demonstrated that these biopolymers offer a wide range of unique applications including preservation of foods from microbial deterioration and formation of biodegradable films.

Alishahi and Aider [172] reported that chitosan films in packaging application tend to exhibit resistance to fat diffusion and selective gas permeability. But, inconvenience comes from their low resistance to water and water vapor transmission. This behavior is due to the strongly hydrophilic character mainly of chitosan, a property that leads to high interaction with water molecules [173]. For this reason, polymer blending or the use of biocomposites and multilayer systems are potential approaches to prepare chitosan-based bioactive coatings.

### 4.1. Antimicrobial Activity

Chitosan was shown to have several advantages over other disinfectants, as it possesses a higher antimicrobial activity, a broader spectrum of activity, a higher kill rate, and lower toxicity towards mammalian cells [174,175]. Many studies have demonstrated that chitosan have an important antimicrobial activity. However, the actual mechanism of inhibition is not yet fully understood. The most feasible hypothesis is a change in cell permeability due to interactions between the positively charged polysaccharide (chitosan at pH lower than 6.5) and the negatively charged membrane. The mechanism underlying the inhibition of bacterial growth should be that the positively charged polymer combines with anionic components such as *N*-acetylmuramic acid, sialic acid and neuraminic acid, on the cell surface.

Firstly, concerning the antibacterial activity of chitosan, possible actions of chitosan and its derivatives have been proposed. Chitosan (especially low-MW particles) could penetrate cell wall of bacteria, combine with DNA and inhibit synthesis of mRNA and DNA transcription [176]. High MW chitosan could interact with cell surface and consequently alter cell permeability [177], or form an impermeable layer around the cell, thus blocking the transport of essential solutes into the cell [178,179]. Chung *et al.* [180] confirmed that antibacterial mechanism includes hydrophilicity and the negative charge of cell surface and the adsorption of chitosan onto the bacterial cell. They show that cell wall hydrophilicity and negative charge of the cell surface were higher in gram-negative bacteria compared to gram-positive and that, in addition, the distribution of negative charges on their cell surfaces was quite different from that of gram-positive. Then, the more negatively charged cell surfaces interact more with positively charged chitosan, in acidic conditions. Their results showed a high value of correlation coefficient between adsorbed chitosan and inhibition efficiency. Moreover, many other studies showed that chitosans are more effective for gram-negative bacteria than gram-positive bacteria [181,182,183].

It was also indicated that adsorbed amounts of chitosan were related to environmental pH values (pH < 6.5) and degree of acetylation of chitosan [183,184,185]. Chitosan is more absorbed by bacterial cells at lower pH in relation with the increase of the chitosan positive ionic charge in relation with the fraction of deacetylated groups (1 − DA). From literature, it is clearly shown that there is a direct relationship between the antibacterial activity of chitosan and its characteristics especially DA. Effect of DA on chitosan antimicrobial activity has been clearly demonstrated in our previous study [186]. In these data, it is clearly shown that the lower DA, the lower MW and the lower pH give the larger efficiency.

In addition, influence of the MW was introduced by Zheng *et al.* [187]. They differentiated the effect of chitosan on *Staphylococcus aureus* (gram-positive) and on *Escherichia coli* (gram-negative) and demonstrated that, for gram-positive *S. aureus*, the antimicrobial activity increases with increase of the molecular weight of chitosan. On the opposite, for gram-negative *E. coli*, they indicate that the antibacterial activity increased with decrease in molecular weight. These authors suggested the two following mechanisms for the antimicrobial activity: in the case of *S. aureus*, the chitosan on the surface of the cell forms a polymeric membrane, which inhibits nutrients from entering into the cell and, for *E. coli*, chitosan with a lower molecular weight entered the cell through pervasion.

Effect of MW was also discussed by Benhabiles *et al.* [188] preparing oligomers of chitin and chitosan. Their antimicrobial activities against four gram-positive and seven gram-negative bacteria were compared to initial chitosan and chitin. They conclude that chito-oligomers would have advantages as new antimicrobial agents due to their higher activity and larger water solubility than the native polysaccharides.

Concerning the antifungal activity, it has been reported that chitosan can reduce the infection of *Fusarium oxysporum* f. sp. apii in celeryand inhibits the spread of *Sphaerotheca pannosa* var. rosae, *Peronospora sparsa* and *Botrytis cinerea* on roses [189,190,191]. Treating tomato plants with chitosan solution reduced mycelial growth, sporangial production, release of zoospores and germination of cysts of *Phytophthora infestans* which resulted in significant disease protection [192]. In addition, chitosan seed treatment could reduce *Colletotrichum* sp. infection and improve performance of chilli seedling [193]. Concerning the mechanism, it is suggested that chitosan forms a permeable film at interface [194] and has two functions: direct interference of fungal growth and activation of several defense processes. These defense mechanisms include accumulation of chitinases, synthesis of proteinase inhibitors, lignification and induction of callous synthesis [195].

In fact, dependence of activities on chitosan characteristics was reported to depend on the particular fungal species. For example, it was shown that fungal growth decreased with increasing MW for *F. oxysporum* and with decreasing DA for *Alternaria solani*, but no MW or DA dependences were observed with *A. niger* [186].

### 4.2. Antioxidant Activity

Oxidative stress belongs to the main causes of many diseases, mainly cancer and cardiovascular problems, which significantly increase the worldwide mortality [196,197,198,199,200,201,202]. Dietary antioxidants, which inactivate reactive oxygen species and provide protection from oxidative damage [196,197,198,199,200,201,202], are considered as important preventive strategic molecules.

Once the lipid oxidation occurs in food products, off-flavors and undesirable chemical compounds are formed and this may be dangerous for health. Therefore, in order to minimize this risk, some antioxidants (like synthetic antioxidants butylated hydroxyanisole (BHA), butylated hydroxytoluene (BHT), t-butylhydroquinone (TBHQ) and propyl gallate) are added to delay the deterioration, caused by lipid oxidation. However, these antioxidants create potential health hazards, and their use has been restricted in some countries. Therefore, there has been a growing interest in natural antioxidants rather than in synthetic ones.

In recent years, much more attention has been paid to study the antioxidant activity of chitosan and its derivatives [203]. It was reported that chitosan and its derivatives act as antioxidants by scavenging oxygen radicals such as hydroxyl, superoxide, alkyl as well as highly stable DPPH radicals tested *in vitro* [204]. Sun and collaborators [205] reported that chitosan and their derivatives act as hydrogen donors to prevent the oxidative sequence.

Furthermore, it was observed that the radical scavenging properties of chitosans depend on their DA and MW. Park *et al.* [204] demonstrated that low-MW chitosans are more active than those with higher MW. Chitosan samples with low MW (1∼3 kDa) revealed higher potential to scavenge different radicals. Other examination showed that low-MW chitosans can exhibit more than 80% of superoxide radical scavenging activity at 0.5 mg/mL concentration [206]. The influence of chitosan MW (30, 90, and 120 kDa) on the antioxidant activity in Salmon skin was also studied [207]. The results of this study showed that all chitosans present antioxidant activities, which reduce Salmon lipid oxidation, the 30-kDa chitosan sample having the higher antioxidant activity. In addition, highly deacetylated (90%) chitin are more preferable for scavenging DPPH, hydroxyl, superoxide and carbon-centered radicals [208]. Even though the precise mechanism of radical scavenging activity is not clear, it is attributed to amino and hydroxyl groups (attached to C-2, C-3 and C-6 positions of the pyranose ring) reacting with unstable free radicals, which facilitate formation of stable macromolecule radicals.

### 4.3. Antitumor Activity

Chitosan and its derivatives possess also antitumor activities investigated by both *in vitro* and *in vivo* method [209]. Some *in vivo* studies reported that chitosan inhibits the growth of tumor cells by exerting immunoenhancing effects. They concluded that the observed antitumor activity was not due to a direct killing of tumor cells, but by an increase of lymphokines production resulting in proliferation of cytolytic T-lymphocytes [210]. Chen *et al.* [211] demonstrated that intratumoral administration of a chitosan gel in animals reduces metastatic breast cancer progression. Chitosan also stimulates macrophages maturing into cytotoxic macrophages and suppresses tumor growth in mice [212]. It has been suggested that elevated secretion of IL-1 and IL-2 causes the anti-tumor effect through maturation and infiltration of cytolytic T-lymphocytes [213].

Other studies demonstrated that chitosan also exhibits a direct effect on tumor cells; it inhibits tumor cell proliferation by inducing apoptosis [214]. For instance, Hasegawa *et al.* [215] showed that chitosan may cause apoptotic death of bladder tumor cells via caspase-3 activation. Further studies revealed that chitosan nanoparticles could also induce necrotic death, which had been tested on liver cancer cells via neutralization of cell surface charge, observed as a decrease in mitochondrial membrane potential and induction of lipid peroxidation [216]. Moreover, chitosan may inhibit Ehrlich ascites tumor growth by reduction of glycolysis causing a decrease in glucose uptake and ATP level in the tumor cells [217]. This study also found that chitosan administered orally at a dose of 1 mg kg^−1^ in mice reduces tumor growth by ~62%, without any toxicity to the liver. Thus, chitosan *per se* possesses potential activity against cancer, even when it is administered orally. A similar study, using a chemically-induced tumor model, showed that addition of chitosan to the diet enables to suppress aberrant crypt tumor lesions in the colon of mice [218]. Interestingly, such protection with chitosan additive in feed lasts only up to 6 weeks. This investigation highlighted that chitosan is responsible for an increase in expression of p21/Cip and p27/Kip and consequently a decrease of expression of proliferating cell nuclear antigen in a human gastric cancer cell line. Nevertheless, further studies are required in order to better understand all mechanisms involved in chitosan-based tumor stasis.

Chitosan activity depends not only on the chitosan structural characteristics, such as DA and MW, but also on tumor species. Jeon and Kim [219] studied the antitumor activity of chitosan oligosaccharides with different molecular weights. They found that chitosan oligosaccharides with MW ranging from 1.5 to 5.5 kDa may effectively inhibit the growth of Sarcoma 180 solid (S180) or Uterine cervix carcinoma No. 14 (U14) tumor in BALB/c mice. Studies on mice examining chitosan samples with different MW revealed significant antimetastatic effects of chitosan against Lewis lung carcinoma. It was shown that the activity increases with decreasing the molecular sizes suggesting an immunostimulating effect which activates peritoneal macrophages and stimulates non-specific host resistance. It was also shown that chitosan samples with higher MW exhibits lower antitumor activity [220]. However, other authors found that decrease of MW of chitosan from 213 to 10 kDa does not affect its *in vitro* cytotoxicity on human lung carcinoma cell line A549 [221]. Additionally, chitosan samples with different MW ranging from 42 to 135 kDa were also evaluated in terms of their cytotoxicity on human bladder cancer RT112 and RT112cp cells and no effect of MW was observed [222]. The same study attempted to examine also the effect of chitosan DA (homogeneous chitosans with DA ranging from 2% to 61%) on cytotoxicity. The results from this experiment indicated that all chitosan samples were active on bladder carcinoma cells, with better activity for samples with higher DA.

It has been shown that chitosan reveals anticancer activity, thus it may be used for encapsulation of anticancer agents. However, before its incorporation into new drugs, pre-tests are required.

## 5. Pharmaceutical and Biomedical Applications of Chitin and Chitosan

The main properties of chitin and chitosan, applied for specific applications, have been already described such as: biocompatibility, renewable origin, non-toxicity [223], non-allergenicity and biodegradability in the body [224]. In addition, due to their attractive biological activities (antifungal, antibacterial, antitumor, immunoadjuvant, antithrombogenic, anticholesteremic agent) and bioadhesivity (especially of chitosan and its derivatives [225]), they are widely used as absorption promoters and hydrating agents, as well as for film production and wound healing [1,2,3,4,226]. Chitin and more easily chitosan may be processed, depending on the intended application, into different conformations such as fibers, powders, films, sponges, beads, solutions, gels and capsules [171]. Consequently, chitosan may be used in oral, nasal as well as ocular routes, for drug delivery in both implantable and injectable forms. Chitin and chitosan in fiber or film state, are mainly applied for tissue engineering and wound care dressing [227,228,229]. Additionally, transmucosal absorption promoter effect of chitosan is especially important for nasal and oral delivery of polar drugs to administrate peptides and proteins and for vaccine delivery [230,231]. Cationic chitosan may affect transport of ions through an interaction with the cell surface (inducing perturbation of membrane phospholipids bilayers). Chitin is also used as excipient and drug carrier in film, gel or powder form for applications involving mucoadhesivity [52]. Actually, the main promising developments are aimed to pharmaceutical and biomedical domains [232,233,234,235,236,237,238,239,240,241,242,243,244,245]. Several selected applications for chitin and chitosan will be described below and summarized in Table 3.

Applications of chitin are less developed compared to those of chitosan due to its large insolubility and difficulties in processing. Therefore, chitin is very often combined with chitosan which gives in fact similar applications.

Chitin accelerates wound-healing in spray, gel and gauze [246,247,248,249]. It is used as support of medicaments or to control drug release [238] taking into account the biodegradability, low toxicity, physiological inertness, antibacterial properties, hydrophilic character, gel forming properties, affinity for proteins and mucoadhesivity [250].

Great attention had been paid to a composite material made of hydroxyapatite-chitin-chitosan which may be used as bone filling material for guided tissue regeneration (treatment of periodontal bony defects). This composite forms a self-hardening paste [251,252,253,254,255,256,257,258,259,260,261]. Chitin was also used for enzymes and whole cells immobilization [262] as well as for tissue engineering [263,264].

Chitosan (the only pseudo-natural polycationic substance) and its electrostatic complexes formed with synthetic or natural polymers (as alginate) are used as antithrombogenic materials for: controlled release, drugs encapsulation, enzymes and cells immobilization and also as gene carriers. Advantage of chitosan-based materials is related to their biodegradability, antibacterial activity, hydrophilic property, as well as presence of polar groups which are able to form secondary interaction with other polymers (-OH and -NH_2_ groups involved in hydrogen bonds and the *N*-acetyl groups in hydrophobic interactions).

**Table 3:** Main applications of chitin and chitosan in pharmaceutical and biomedical domains.

Forms	Applications
Beads	Drug delivery [266]
Microspheres [265]	Enzyme immobilization
	Gene delivery vehicle [267]
Nanoparticles	Encapsulation of sensitive drugs [172]
Coatings	Surface modification
	Textile finishes
Fibers	Medical textiles
	Suture
Nanofibers [268]	Guided bone regeneration
	Scaffold for nerve tissue regeneration
Nonwonen bioactive fibers [269]	Wound healing
Films	Wound care
	Dialysis membrane
	Antitumoral [270]
	Semi-permeable film for wound dressing [271]
Powder	Adsorbent for pharmaceutical and medical devices
	Surgical glove powder
	Enzyme immobilization
Sponge [272]	Mucosomal hemostatic dressing
	Wound dressing
	Drug delivery [272]
	Enzyme entrapment
	Artificial skin [271]
Shaped objects	Orthopedics
	Contact lenses
Solutions	Cosmetics
	Bacteriostatic agent
	Hemostatic agent
	Anticoagulants
	Antitumor agent
	Gene delivery [267]
	Spermicide [245]
Gels	Delivery vehicle
	Implants, coating
	Tissue engineering
	Wound dressing for wet treatment [271]
Tablets	Compressed diluent
	Disintegrating agent
	Excipient [273]
Capsules	Delivery vehicle

Materials for wound dressing and tissue engineering are important but still under development [274,275,276,277,278,279,280,281,282]. New adhesives were also proposed [283,284]. The Az-chitosan derivative is non-toxic, cytocompatible and mechanically suitable for peripheral surgeries [285]. Chitosan films, like many other polysaccharide-based films, exhibit resistance to fat diffusion and selective gas permeability but they are relatively poor in terms of resistance to the transmission of water and water vapor. This behavior is observed due to their hydrophilic character leading to high interaction with water molecules [173]. In order to overcome this problem, polymer blending or biocomposites and multilayer systems are used for preparation of chitosan-based bioactive and stable coatings.

Mucoadhesivity of chitosan and its cationic derivatives is recognized and proved to enhance the adsorption of drugs especially at neutral pH. *N*-trimethyl chitosan chloride interacts with the negatively charged cell membranes [286]. *N*-lauryl-carboxymethylchitosan being an amphiphilic polymer forms micelles solubilizing taxol which becomes more efficient. This type of chitosan derivative is safe in terms of membrane toxicity and it could be useful as carrier for hydrophobic cancer drugs [287,288]. Chitosan or its derivatives were used for gene transfection. It was shown for *N*-alkylated chitosan that transfection efficiency increases upon elongation of the alkyl side chains up to eight carbons in the side chain [289]. Quaternized chitosan was also used for the same purpose [290]. Porous chitosan (and derivatives) microspheres were prepared in order to deliver antigens in a controlled way [291]. This type of particles was loaded with Newcastle disease virus vaccine and tested *in vitro* and *in vivo* [291,292].

An interesting application of the chitosan- calcium phosphate cement was found. Chitosan or chitosan glycerophosphate was mixed with calcium phosphate and citric acid and an attractive injectable self-hardening system for bone repair or filling indications was formed [253,254,255].

At the end, several examples of applications for drug delivery are mentioned [265,293,294,295,296]. Chitosan may be processed more easily than chitin to different forms: in sponge, capsule or nanoparticle depending on the tested system and the goal of its administration.

## 6. Conclusions

In this review, the characteristics of chitin and chitosan are described. This was followed by a discussion on the solubilization required to process the polysaccharides to obtain new materials. Their fiber and film-forming abilities are recognized on the basis of the H-bonds network formation in the solid state which may be useful for new potential applications.

However, the most important applications come from their hydrophilic character and antimicrobial properties, especially desired for production of new biomaterials.

Chitosan in comparison with chitin is soluble in acidic media, which is applied for improvement of processing methods. In fact, chitosan may be easily processed as fiber, film, sponge, bead, gel or solution. Additionally, its cationic charge provides the possibility to form electrostatic complexes and/or multilayer structures. The presence of free -NH_2_ groups along chitin and chitosan chains allows to perform specific modifications (performed on the C-2 position of the D-glucosamine unit) under pretty mild conditions (even in aqueous conditions with chitosan). Furthermore, chitin and chitosan can be blended with synthetic or natural polymers (proteins, DNA, alginate, hyaluronan, *etc.*).
